# Development and applicability assessment of a 5R-principle-guided evaluation system for antimicrobial stewardship in open extremity trauma using a hybrid Delphi–analytic hierarchy process model

**DOI:** 10.3389/fcimb.2026.1805356

**Published:** 2026-07-21

**Authors:** Jiayu Deng, Shuyu Chen, Chen Zhou, Xuezheng Li, Shenghong Wu, Na Liu, Tianzi Lei, Yanqing Song

**Affiliations:** 1 Department of Pharmacy, Lequn Branch, The First Hospital of Jilin University, Changchun, China; 2 College of Pharmacy, Yanbian University, Yanji, China; 3 General Department, The First Hospital of Jilin University (the Eastern Division), Changchun, China; 4 Department of Pharmacy, The Affiliated Hospital of Yanbian University (Yanbian Hospital), Yanji, China; 5 Department of Pharmacy, Jilin Province First Automobile Works (FAW) General Hospital, Changchun, China; 6 Department of Pharmacy, Tonghua Central Hospital, Tonghua, China; 7 School of Pharmaceutical Sciences, Jilin University, Changchun, China

**Keywords:** analytic hierarchy process, antimicrobial stewardship, appropriate antimicrobial use, Delphi method, open extremity trauma

## Abstract

**Background:**

Open extremity trauma is associated with a high risk of infection, making appropriate antimicrobial use essential. However, existing stewardship tools are often generic and lack a structured, hierarchical evaluation system tailored to this setting.

**Methods:**

We developed an initial indicator set based on the 5R principles (Right Patient, Right Drug, Right Dose, Right Time, and Right Route). A national panel of 42 experts participated in two rounds of a modified Delphi survey using a 5-point Likert scale to rate indicator importance. The analytic hierarchy process (AHP) was used to assign indicator weights. The finalized system was preliminarily assessed in a retrospective cohort from the Hand and Foot Surgery Department at the Lequn Campus, First Hospital of Jilin University, comparing a pharmacist-led intervention group with a routine-care control group.

**Results:**

Response rates were 100% in both Delphi rounds, and the expert authority coefficient was 0.87. Across the two rounds, Kendall’s W values ranged from 0.044 to 0.110 for secondary and tertiary indicators, with all P < 0.05, demonstrating statistically significant expert agreement. All indicators met predefined consensus criteria (mean score ≥ 4.0; coefficient of variation ≤ 0.25). The final system comprised 5 primary, 11 secondary, and 22 tertiary indicators; all AHP consistency ratios were < 0.1. In the preliminary applicability assessment, the intervention group achieved higher appropriateness scores (P=0.004) and lower total antimicrobial costs (P<0.001) than the control group.

**Conclusion:**

A 5R-guided, Delphi–AHP-derived evaluation system was developed and preliminarily validated. The tool can support standardized assessment and targeted pharmacist-led antimicrobial stewardship in open extremity trauma.

## 1 Introduction

Trauma is a major global health challenge. In 2019, trauma-related deaths worldwide totaled 4.3 million, representing a substantial component of the global disease burden (GBD 2019 Diseases and Injuries Collaborators, 2020). Open extremity injuries are common and carry a high risk of bacterial contamination because wounds are directly exposed to the external environment, with reported infection rates of 15%–30%. Infections can delay wound healing and lead to severe complications such as sepsis and osteomyelitis, potentially resulting in limb dysfunction and increased disability and mortality.

Appropriate antimicrobial use is central to infection prevention and treatment in open extremity trauma and directly influences clinical outcomes. Yet, prescribing in practice is often suboptimal, including unclear indications that prompt unnecessary broad-spectrum use, inappropriate empirical agent selection, and prolonged treatment durations. These practices accelerate antimicrobial resistance and increase the burden of drug-resistant infections (Sakeena et al., 2019). They also raise direct medical costs and impose a substantial economic burden on healthcare systems (Zhou et al., 2023).

Although national and international guidelines provide recommendations for antimicrobial prophylaxis and treatment in open fractures and related injuries, adherence in real-world practice remains inconsistent. Antimicrobial stewardship programs (ASPs) aim to close this gap by optimizing antimicrobial use, improving patient outcomes, and reducing costs associated with healthcare-associated infections (van den Bosch et al., 2015; Schuts et al., 2016; Wagner et al., 2014). Multiple studies show that clinical pharmacist involvement can improve antimicrobial selection and other key prescribing behaviors (Kooda et al., 2022; Zhou et al., 2015). A perioperative systematic review and meta-analysis reported that pharmacist-led stewardship programs improve antimicrobial appropriateness, adherence to dosing schedules, and treatment duration, and reduce surgical site infections (Naseralallah et al., 2024). However, pharmacist-led stewardship research in open extremity trauma is limited, and standardized evaluation tools specific to this population are lacking. This gap limits timely identification of drug-related problems and targeted interventions.

The 5R principles (Right Patient, Right Drug, Right Dose, Right Time, and Right Route) provide a coherent framework for antimicrobial stewardship decisions (Cheng et al., 2023). However, in many studies the 5R principles are used only to categorize high-level domains, without being translated into operational, measurable, and weighted secondary and tertiary indicators. This limits their utility for standardized assessment and continuous quality improvement.

The Delphi method is a structured group decision-making technique that synthesizes multidisciplinary expert opinions through iterative anonymous surveys to reach consensus. It is widely used to develop evaluation indicator systems in healthcare (Yao et al., 2024; Price et al., 2020; Wu et al., 2026). As a multi-criteria decision-making method, the analytic hierarchy process (AHP) can quantify the relative importance of indicators through pairwise comparisons and consistency checks (Guan et al., 2024). Combining Delphi and AHP has proven useful for constructing evidence-informed medication evaluation systems in other populations (Lin et al., 2020), but has not been systematically applied to antimicrobial use in open extremity trauma.

Therefore, this study aimed to develop a multidimensional, hierarchical, and weighted evaluation system for appropriate antimicrobial use in open extremity trauma under the 5R framework using a hybrid Delphi–AHP approach. We further conducted a preliminary applicability assessment to explore whether pharmacist-led interventions guided by this system improve appropriateness scores and reduce antimicrobial costs.

## 2 Methods

**Figure 1:**
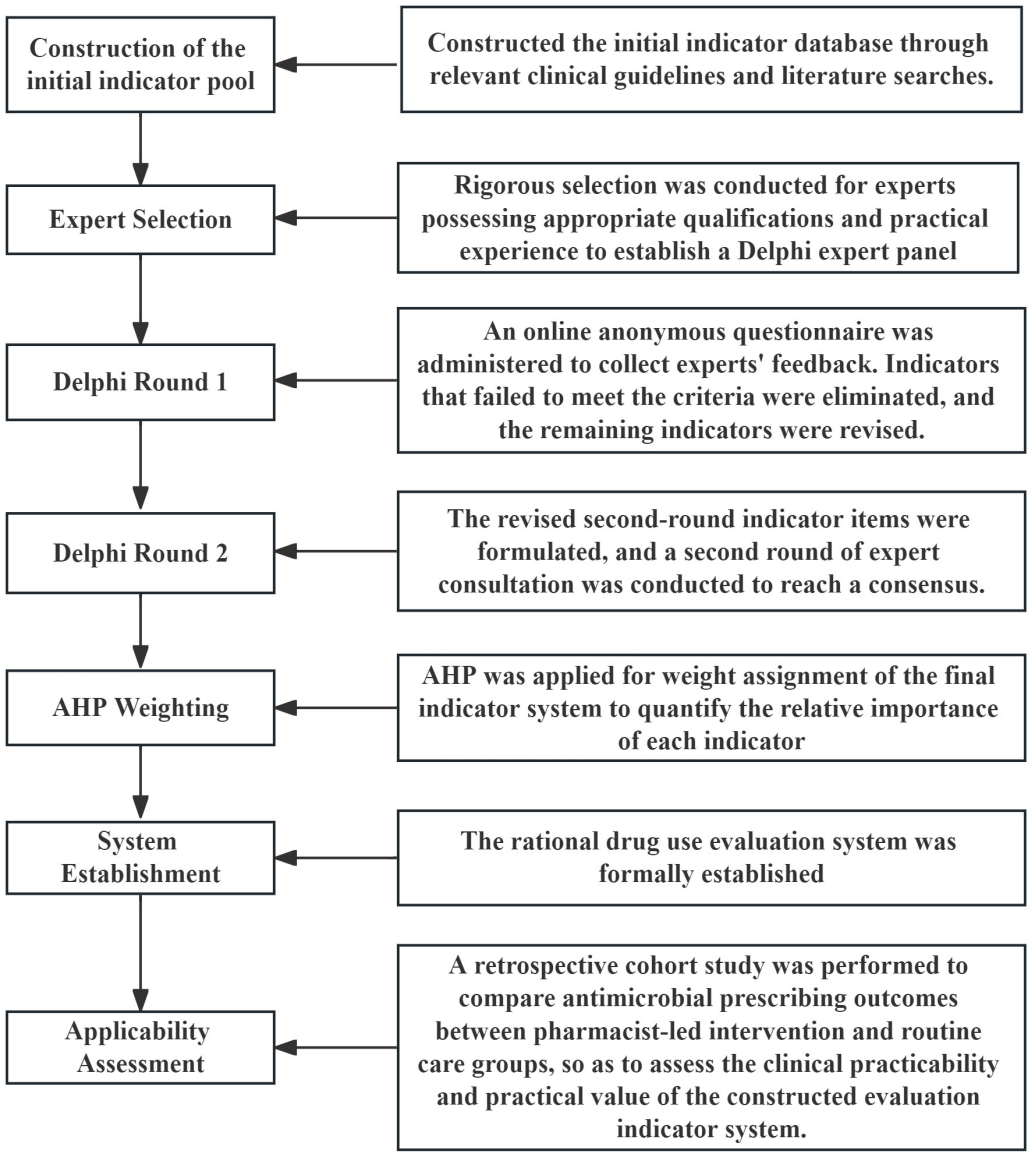
Study workflow for developing and preliminarily validating the evaluation system for appropriate antimicrobial use in open extremity trauma (submitted as a separate figure file).

This study followed the Guidance on Conducting and REporting DElphi Studies (CREDES) (Jünger et al., 2017). We first reviewed relevant guidelines and literature to generate a preliminary framework and candidate indicators for evaluating appropriate antimicrobial use in open extremity trauma. We then conducted two rounds of anonymous, web-based Delphi consultation in July and August 2025 to refine indicators and reach expert consensus using a 5-point Likert scale. The analytic hierarchy process (AHP) was applied to derive indicator weights and establish a weighted evaluation system. Finally, we conducted a retrospective cohort study to assess the system’s real-world applicability power. Importantly, this evaluation tool was applied retrospectively to pre-existing cohorts. During the intervention period (2023–2024), clinical pharmacist-led therapy pathways were guided by general stewardship principles, not by this newly developed tool. The study workflow is presented in **Figure 1**.

This study was conducted in accordance with the Declaration of Helsinki and approved by the Institutional Review Board of the First Hospital of Jilin University (Approval No. 2025-370). Because this was a retrospective study using data extracted from the electronic medical record system, the requirement for written informed consent was waived.

### 2.1 Development of the rational medication evaluation system

#### 2.1.1 Construction of the initial indicator pool

We based our approach on the consensus of authoritative experts, with a primary focus on the clinical application and management of antimicrobial agents (Chinese Medical Association, 2015; Trauma Branch of Chinese Medical Association, 2016; Trauma Orthopaedic Group, 2014). Using keywords such as “Delphi method,” “antimicrobial stewardship,” “rational drug use,” and “drug-related issues,” we searched Chinese and English databases including PubMed, Web of Science, and China National Knowledge Infrastructure (CNKI). This process generated an initial indicator database for evaluating rational antimicrobial use in patients with open extremity injuries.

#### 2.1.2 Selection of the expert panel

The selection of Delphi panel members is crucial for the scientific evaluation of the assessment system’s indicators. To ensure the panel’s authority and representativeness, members must have professional backgrounds in relevant fields and possess in-depth knowledge and clinical experience related to the rational use of antimicrobial agents open extremity trauma. Previous studies indicate that consensus saturation is typically achieved with 11 to 50 experts participating in the Delphi method (Diamond et al., 2014; Obeng et al., 2025). We invited 42 experts from multiple provinces and diverse medical institutions across China to form the Delphi panel, who participated via an online questionnaire. The questionnaire’s introductory page provided a detailed explanation of the study’s specific content.

Expert selection criteria were as follows:

1. Educational background: Bachelor’s degree or higher.
2. Professional title: Representing the full range of professional technical titles to ensure comprehensive perspectives and expertise across hierarchical levels.
3. Work experience: Minimum of 5 years in the relevant field, with proficiency in antimicrobial use and the ability to assess its appropriateness.
4. Communication skills: Clear expression and ability to complete at least two rounds of questionnaire correspondence.
5. No direct conflict of interest related to the study.

#### 2.1.3 Preliminary questionnaire development

The initial indicator pool was developed through a structured process before the formal Delphi survey. A dedicated researcher independently reviewed relevant clinical guidelines and literature to identify potential items related to appropriate antimicrobial use in patients with open extremity trauma. These items were then synthesized and organized by a clinical pharmacist on the research team with extensive expertise in hand and foot surgery, forming a draft indicator list. Following group discussions within the research team, the pharmacist revised the list and drafted the preliminary Delphi questionnaire. The evaluation system explicitly adopted the 5R principles as primary indicators, establishing a systematic analytical framework through clear categorization of secondary indicators. The preliminary questionnaire comprised 5 primary indicators, 12 secondary indicators, and 22 tertiary indicators (**Supplementary Table 1**).

#### 2.1.4 Data collection

During the two Delphi rounds, experts rated the necessity and importance of each indicator and self-assessed their authority (authority coefficient Cr) (Brown, 1968). Kendall’s coefficient of concordance (W) (Chen et al., 2016) was calculated from the questionnaire data to quantify the degree of agreement among experts for each indicator. It ranges from 0 to 1, with higher values indicating stronger consensus. The experts’ positive coefficient served as a measure of engagement, which is reflected by the questionnaire response rate. Higher rates generally correspond to greater expert involvement and more reliable outcomes (Taherdoost and Madanchian, 2024). To maintain a high response rate, timely reminders were sent to panel members during each survey round.

The expert authority coefficient, which reflects an expert’s familiarity with the evaluation indicators, was calculated based on the judgment criteria (Ca) and the familiarity coefficient (Cs) (Jiang et al., 2020). Ca comprised four dimensions: theoretical basis, clinical practice experience, domestic and international literature, and intuitive perception. Cs consisted of five levels, quantified as familiarity scores ranging from 0.1 for “not familiar” to 0.9 for “very familiar” (**Supplementary Tables 2**, **3**).

#### 2.1.5 Delphi rounds and item screening

##### 2.1.5.1 First round

An online questionnaire detailing the research was sent to experts at the end of July 2025. The first page provided a study description and collected experts’ demographic and professional information. The main body of the questionnaire used a 5-point Likert scale (1 = very unimportant, 5 = very important). An open-ended text box followed each tier of indicators, allowing experts to offer comments or suggestions (Zhang et al., 2022; Preston and Colman, 2000).

After the first Delphi round, the mean score, coefficient of variation (CV), and maximum score ratio (Kj) were calculated for each indicator. Indicators meeting any of the following criteria were excluded (Feng, 2018): (1) mean < 4.0; (2) CV > 0.25; (3) Kj ≤ 30%. For indicators that failed to meet the exclusion criteria and were retained, the research team revised and optimized them based on feedback from the expert panel. All revised indicators were included in the second-round questionnaire. As the core framework, the five primary indicators remained fixed and were not included in the second-round consultation.

##### 2.1.5.2 Second round

The revised questionnaire was submitted to panel members who completed the first round for reassessment of importance, familiarity, and judgment basis. Items with a mean score of at least 4.0 and a coefficient of variation ≤ 0.25 were considered to have reached consensus (Zhao et al., 2024). The positive coefficient, authority coefficient, and Kendall’s coefficient of concordance (W) were calculated. Based on the feedback from this round, the evaluation system was further refined.

##### 2.1.5.3 Data analysis in the Delphi method


Mean Score=(Sum of all item scores)/(Number of items)

Coefficient of Variation (CV)=(Standard deviation of item scores/Mean score of items)×100%

Percentage of Maximum Scores (Kj)=(Number of experts assigning the maximum score to an item/Total number of experts)×100%

Positive Coefficient of Experts=(Number of returned questionnaires/Number of questionnaires distributed)×100%

Authority Coefficient (Cr)=(Ca+Cs)/2

Kendall’s Coefficient of Concordance (W) was used to represent the degree of expert agreement, and the Chi-square (χ²) test was applied to assess differences. A p-value < 0.05 was considered statistically significant.

#### 2.1.6 AHP for weight determination

The AHP method was used to assign weights to each indicator. Based on Saaty’s 1–9 scale (**Supplementary Table 4**), pairwise comparisons were conducted to construct judgment matrices. Relative weights were derived using the geometric mean method, followed by a consistency check. When the consistency ratio (CR) exceeded 0.1, experts were asked to re-evaluate until all matrices achieved CR < 0.1, ensuring reliability. After a satisfactory consistency check, the elements in each column were normalized to compute the final local weights. To determine global weights, the cumulative influence of weights from higher-level indices was incorporated (Di et al., 2022). The global weight of an indicator was calculated as the product of its local weight and the local weights of all its parent indicators across the hierarchy.

### 2.2 Preliminary applicability assessment of the rational drug use evaluation system

A pilot study was conducted in the Hand and Foot Surgery Department at Lequn Campus, First Hospital of Jilin University, to validate the clinical applicability of the evaluation system. The Department Qualification Statement is provided in **Supplementary Material 2**.

#### 2.2.1 Inclusion and exclusion criteria

Inclusion Criteria: Patients of all ages (including pediatric and adult patients) admitted with open extremity trauma, including open extremity wounds, open fractures, or joint injuries.

Exclusion Criteria: (1) Poor adherence to or refusal of the treatment plan. (2) Patients who did not undergo surgery (3) Patients transferred to other departments during hospitalization. (4) Patients with incomplete medical records.

#### 2.2.2 Sample size calculation

The primary outcome was the appropriateness of antimicrobial use. Previous research reported a pre-intervention rate of 1.3%, which increased significantly to 12.4% following an effective pharmacist-led intervention (Butt et al., 2019). Using G Power 3.1 software with a two-tailed α = 0.05, 80% power, and a 1:1 allocation ratio, the minimum required sample size was calculated as 86 cases per group (total n = 172). To ensure adequate statistical power and reliability, 100 cases per group were enrolled.

#### 2.2.3 Patient grouping, intervention, and evaluation metrics

The study retrospectively identified eligible subjects through the hospital’s electronic medical record system and divided them into two temporal cohorts based on their admission dates. Patients were selected from the initial candidate pool using simple random sampling with a random number table generated by SPSS 27.0 software as the final subjects for analysis.

Control Group: Patients admitted between August 1, 2022, and July 31, 2023, were identified. Following the inclusion and exclusion criteria, 391 eligible patients formed the initial candidate pool. Then, 100 of them were randomly selected for the study. This group received routine antimicrobial stewardship without pharmacist-led structured interventions.

Intervention Group: Patients admitted between October 1, 2023, and September 30, 2024, who received systematic pharmacist interventions were identified. After screening using the same criteria, a candidate pool of 497 patients formed the intervention group, from which 100 were randomly selected for inclusion in the study. During the intervention period, patients received a pharmacist-led “Five-Component Standardized Medication Therapy Pathway”. This pathway included: (1) daily ward round interventions; (2) prescription review and optimization; (3) continuous monitoring of safety and pathogen results; (4) routine education and training; (5) case consultation and discussion (**Supplementary Material 2**). This standardized pathway ensured consistency of the intervention across all patients in the intervention group.

Primary outcome measures was the “Rational use score of antibacterial drugs,” calculated using the novel evaluation system. Each patient’s rationality score was derived by applying the global weights of the system’s indicators to a 100-point scale, with each item individually assessed against the patient’s medical records. The detailed operational scoring criteria for each indicator are provided in **Supplementary Material 2**. Secondary outcome measure was the “Total antimicrobial cost.” It was based on the drug cost recorded in the hospital’s electronic medical record system. It reflected the actual acquisition cost without any additional markup. Data were collected by independent research assistants blinded to group allocation. During data extraction, baseline liver and renal function status were determined based on the first blood test results obtained after hospital admission. Liver function was categorized as abnormal if any of the following parameters exceeded the institutional laboratory reference range: ALT (> 50 U/L), AST (> 40 U/L), or total bilirubin (> 20.5 μmol/L). Renal impairment was defined as an eGFR ≤ 60 mL/min/1.73m².

#### 2.2.4 Statistical analysis

Data were managed and analyzed using Microsoft Excel 2021 and SPSS 27.0. Continuous data with a normal distribution are presented as mean ± standard deviation (x̄ ± s), while non-normally distributed data are expressed as median (interquartile range) [M (Q1, Q3)]. Between-group comparisons were conducted using the independent samples t-test for normally distributed variables and the Mann-Whitney U test for non-normally distributed variables. Categorical variables were analyzed using the chi-square test. A two-sided P-value < 0.05 was considered statistically significant. Subgroup analyses of the five primary indicator dimensions were performed using the same methods. Inclusion and exclusion criteria ensured completeness of key variables; cases with missing data were excluded during initial data entry.

## 3 Results

### 3.1 Expert characteristics

All 42 invited experts completed both Delphi rounds, yielding a 100% response rate and indicating high engagement. The expert panel consisted of clinical pharmacists and related specialists from multiple tertiary hospitals across six provinces and 12 cities in China, representing broad geographic coverage. Detailed information is provided in **Supplementary Table 5**. Most experts held postgraduate degrees, had more than 10 years of clinical experience, and occupied senior professional titles (**Table 1**).

**Table 1:** Baseline characteristics of the Delphi expert panel.

Characteristics		Number	Percentage (%)
Gender	Male	20	47.62
	Female	22	52.38
Age, y	<30	2	4.76
	≥30, <40	13	30.95
	≥40, <50	22	52.38
	≥50	5	11.90
Highest level of education	Bachelor	9	21.43
	Master	24	57.14
	Doctorate	9	21.43
Professional title	Senior	30	71.43
	Intermediate	10	23.81
	Primary	2	4.76
Work experience, y	5-10	7	16.67
	11-15	16	38.10
	>15	19	45.24
Current area of work	Clinical Pharmacy	22	52.38
	Orthopaedics	8	19.05
	Hand and Foot Surgery	5	11.90
	Infection Prevention and Control Department	2	4.76
	Medical Affairs Department	5	11.90

Professional titles are defined according to the standardized Chinese healthcare professional title system. Senior: Chief or Associate Chief Physician/Pharmacist; Intermediate: Attending Physician/Pharmacist; Primary: Resident Physician/Junior Pharmacist.

### 3.2 Delphi round results

#### 3.2.1 First round

In the first Delphi round, no indicator had a mean score < 4.0 or a maximum-score ratio (Kj) ≤ 30% (**Supplementary Table 6**). Within Theme B (Right Drug), the tertiary indicator “B4.1: Whether the drug specification matches the prescribed dose in the medical order” was removed because its coefficient of variation exceeded the prespecified threshold (CV = 0.29). After removal, the corresponding secondary indicator (B4) no longer provided independent evaluative value; its content was integrated into Theme C (Right Dose), under “C1 Implementation of routine dosage,” consistent with clinical practice.

In addition, based on the valuable feedback from the expert panel, we revised eight indicators under Themes A, B and D to improve their clarity and standardization, and added one new indicator under Theme E to optimize route-drug matching. Overall, two items under Theme B were deleted; one item under Theme A, five items under Theme B, and two items under Theme D were revised; and one item under Theme E was added (**Table 2**).

**Table 2:** Indicator revisions of the first round.

Clinical question	Mean score	Coefficient of variation (%)	Full-score frequency (%)	Excluded
Theme A: Right Patient				
A1.2 Conducting infection risk assessment for the patient.	4.55	0.11	54.76	NO
Theme B: Right Drug				
B1 Giving priority to first-line drugs recommended by guidelines.	4.55	0.12	57.14	NO
B1.1 Whether the selection of preventive drugs conforms to guideline recommendations.	4.67	0.10	66.67	NO
B2 Guiding medication based on drug sensitivity test results.	4.60	0.19	71.43	NO
B2.2 Whether drugs are selected based on drug sensitivity test results.	4.60	0.19	69.05	NO
B2.3 Whether medications are adjusted in a timely manner based on drug sensitivity test results.	4.50	0.13	54.76	NO
B4 Selecting drugs with appropriate specifications.	4.21	0.23	45.25	NO
B4.1 Whether the drug specification matches the dosage prescribed in the medical order.	4.07	0.29	42.86	YES
Theme D: Right Time				
D2.1 Whether the duration of preventive medication meets the requirements of guidelines.	4.48	0.14	54.76	NO
D2.2 Whether the course of therapeutic medication conforms to the specifications in the instruction manual.	4.33	0.16	45.24	NO

Merged/Revised indicators		
Original Indicator	Action	New Outcome
B4 Selecting drugs with appropriate specifications.	Merged	Integrated into Theme C (Right Dose), under “C1 Implementation of routine dosage”.
A1.2 Conducting infection risk assessment for the patient.	Revised	A1.2 Performing infection risk stratification for the patient.
B1 Giving priority to first-line drugs recommended by guidelines.	Revised	B1 Giving priority to first-line drugs recommended by guidelines or specifications.
B1.1 Whether the selection of preventive drugs conforms to guideline recommendations.	Revised	B1.1 Whether the selection of preventive drugs conforms to the evidence-based recommendations in current guidelines and specifications.
B2 Guiding medication based on drug sensitivity test results.	Revised	B2 Guiding medication based on drug sensitivity or NGS test results.
B2.2 Whether drugs are selected based on drug sensitivity test results.	Revised	B2.2 Whether drugs are selected based on drug sensitivity or NGS test results.
B2.3 Whether medications are adjusted in a timely manner based on drug sensitivity test results.	Revised	B2.3 Whether medications are adjusted in a timely manner if empirical drugs are inconsistent with test results.
D2.1 Whether the duration of preventive medication meets the requirements of guidelines.	Revised	D2.1 Whether the duration of preventive medication meets the requirements of current guidelines and specifications.
D2.2 Whether the course of therapeutic medication conforms to the specifications in the instruction manual.	Revised	D2.2 Whether the course of therapeutic medication meets the requirements of current guidelines and specifications.
Added indicators		
E1.2 Whether the drug matching the medication administration route is selected.		

#### 3.2.2 Second round

Based on the round-1 quantitative results and qualitative feedback, the questionnaire was revised after discussion within the research team. The updated round-2 questionnaire comprised 5 primary, 11 secondary and 22 tertiary indicators. All items met the consensus criteria in round 2 (**Table 3**; **Supplementary Table 7**).

**Table 3:** Evaluation indices of the second round.

Clinical question		Mean score	Coefficient of variation (%)	Full-score frequency (%)	Consensus
Theme A: right patient					
A1	Whether the patient has indications for preventive medication.	4.71	0.12	76.19	YES
A2	Whether the patient has indications for therapeutic medication.	4.67	0.11	69.05	YES
Theme B: right drug					
B1	Giving priority to first-line drugs recommended by guidelines or specifications.	4.62	0.13	66.67	YES
B2	Guiding medication based on drug sensitivity or NGS test results.	4.52	0.16	61.90	YES
B3	Consideration of special circumstances.	4.19	0.18	38.10	YES
Theme C: right dose					
C1	Implementation of routine dosage.	4.33	0.16	45.24	YES
C2	Individualized dosage adjustment.	4.29	0.25	54.76	YES
Theme D: right time					
D1	Medication administration timing.	4.64	0.11	66.67	YES
D2	Medication course.	4.45	0.17	59.52	YES
Theme E: right route					
E1	Selection of medication administration route.	4.48	0.19	61.90	YES
E2	Conversion of medication administration route.	4.12	0.24	40.48	YES

Consensus was defined as a mean score≥4.0 and a coefficient of variation (CV)≤0.25.

### 3.3 Delphi consultation results

#### 3.3.1 Delphi process results

**Figure 2:**
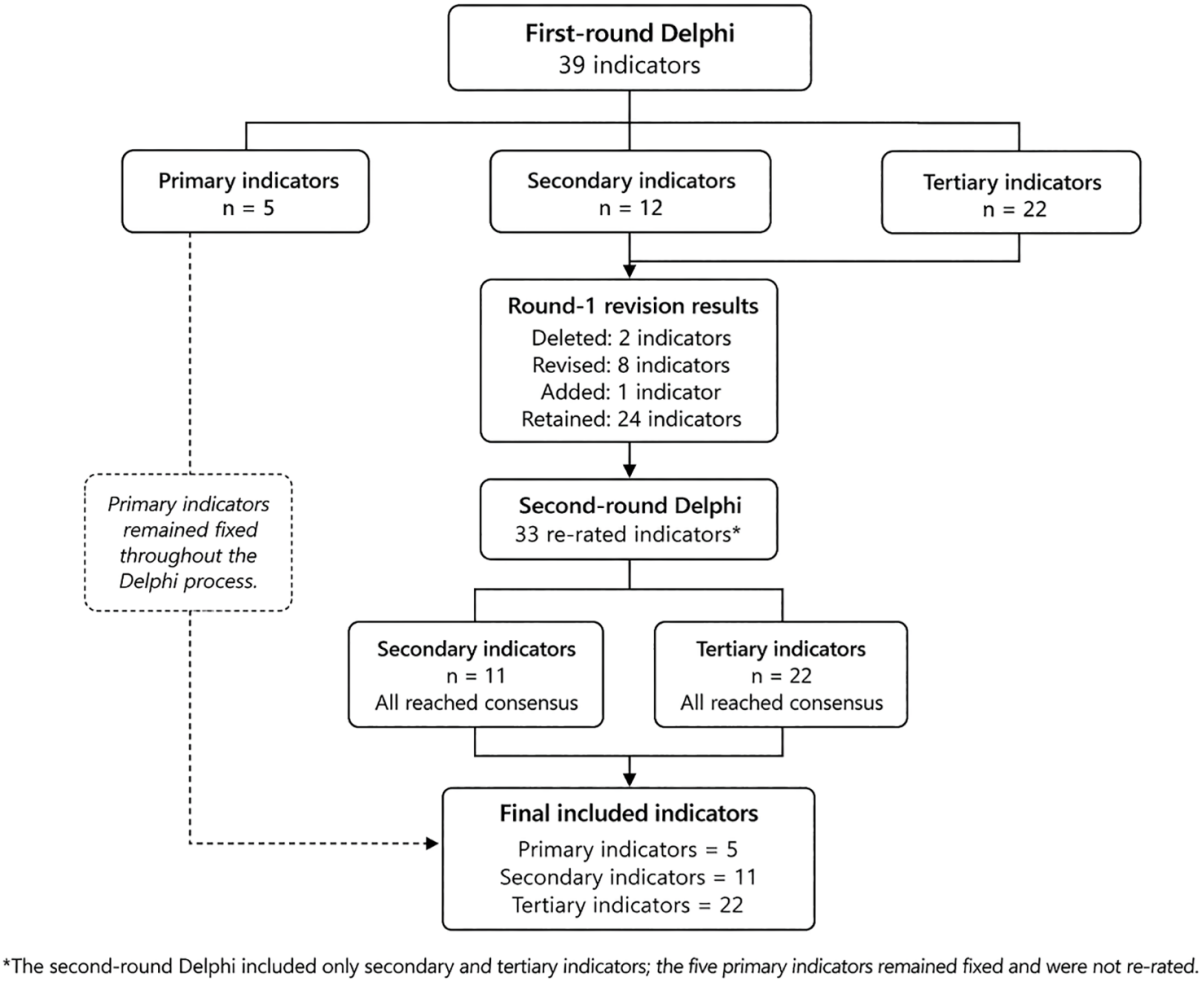
Indicator selection and modification during the Delphi process (submitted as a separate figure file).

**Figure 2** summarizes the Delphi process. The initial survey assessed 39 indicators (5 primary, 12 secondary, and 22 tertiary). The five primary indicators, which constitute the 5R framework, were retained as the fixed structure and were not re-rated in round 2. Among secondary and tertiary indicators, 2 were deleted, 8 were revised, 1 was added, and 24 were retained. After round 2, consensus was achieved on all 33 indicators, resulting in a final system comprising 5 primary, 11 secondary, and 22 tertiary indicators.

#### 3.3.2 Expert authority coefficients

The authority coefficient was 0.87 (87.38%) in the first round and 0.87 (86.55%) in the second. Both exceeded the recognized threshold of 0.7, demonstrating the high credibility and specialized authority of the panel (**Table 4**). The judgment criterion (Ca) and familiarity coefficient (Cs) remained at high levels throughout both rounds, reflecting the experts’ strong theoretical foundation and practical experience regarding the evaluation indicators. This further ensures the scientific rigor and reliability of the indicator revision process.

**Table 4:** Expert authority coefficient (Cr) across two rounds of Delphi consultation.

Delphi round	Ca	Cs	Cr
Round 1	0.96	0.79	0.87
Round 2	0.97	0.76	0.87

Ca, Coefficient of judgment; Cs, Coefficient of familiarity.

#### 3.3.3 Expert agreement and concordance

In round 1, Kendall’s W values for secondary and tertiary indicators were 0.110 and 0.044, respectively; in round 2, they were 0.101 and 0.057. The modest W values likely reflect the multidimensional structure of the indicator system and heterogeneity in expert interpretation. Notably, concordance for tertiary indicators improved after revision. Chi-square tests indicated significant agreement in both rounds (all P < 0.05), supporting convergent expert consensus (**Table 5**).

**Table 5:** Kendall**’**s coefficient of concordance (W) for expert opinion across two Delphi rounds.

Index	Round 1		Round 2	
	Second indicator	Tertiary indicator	Second indicator	Tertiary indicator
Kendall’s W	0.110	0.044	0.101	0.057
χ²	50.911	38.651	42.614	50.024
P	<0.001	0.011	<0.001	<0.001

### 3.4 Weighting of evaluation system indicators

**Figure 3:**
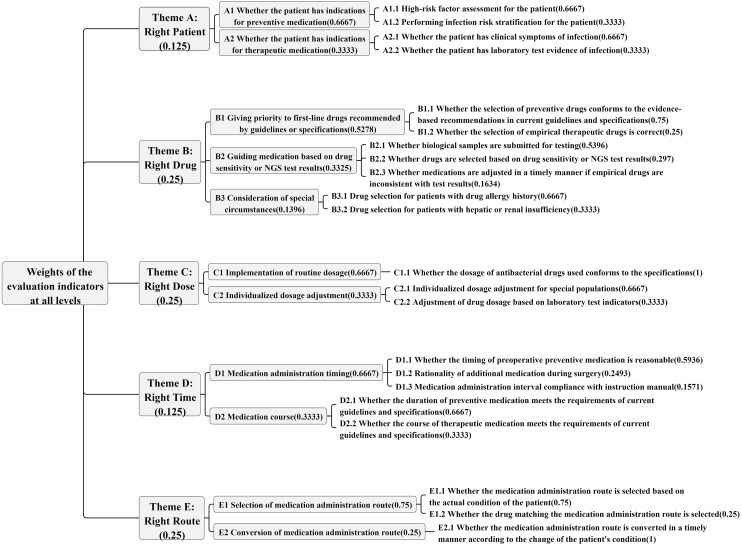
Weights of the evaluation indicators at all levels (submitted as a separate figure file).

Indicator weights were derived using AHP, with higher weights reflecting greater relative importance. After consistency testing, all judgment matrices achieved consistency ratios (CRs) < 0.1, confirming acceptable reliability. Among the five primary dimensions, Right Drug, Right Dose, and Right Route had the highest weights (each 0.25), suggesting experts place greatest emphasis on appropriate agent selection, dose optimization, and route selection. At the secondary level, “E1 Selection of medication administration route” carried the highest weight. At the tertiary level, “B1.1 Whether the selection of preventive drugs conforms to the evidence-based recommendations in current guidelines and specifications” had a relatively high weight, highlighting the importance of guideline adherence (**Figure 3**; **Supplementary Table 8**).

### 3.5 Clinical application of the evaluation system

No significant statistical differences were observed between the two groups in baseline characteristics, including gender, age, liver and kidney function, anesthesia mode, surgical site, and Incision type (all P > 0.05), confirming their comparability (**Table 6**).

**Table 6:** Comparison of baseline characteristics between control and intervention groups.

Characteristic	Control group (n=100)	Intervention group (n=100)	P value
Gender (Male: Female)	80:20	79:21	0.861
Age [M (Q1, Q3)]	45.50 (33.00, 56.00)	45.50 (35.00, 53.75)	0.957
Liver function status (Normal: Abnormal)	87:13	94:6	0.091
Renal function status (Normal: Abnormal)	97:3	96:4	1.000
Anesthesia mode (General anesthesia: Local anesthesia)	44:56	38:62	0.388
Surgical site (Upper Limb: Lower Limb: Multiple Sites)	77:22:1	81:18:1	0.778
Incision type (Class III: Class IV)	98:2	100:0	0.497

Abnormal liver function was defined based on the institutional routine clinical laboratory reference ranges: alanine aminotransferase (ALT) > 50 U/L, aspartate aminotransferase (AST) > 40 U/L, or total bilirubin > 20.5 μmol/L. Abnormal renal function was defined as an estimated glomerular filtration rate (eGFR) ≤ 60 mL/min/1.73m². All baseline laboratory parameters were assessed using the first blood test results obtained after hospital admission.

Clinical applicability assessment showed that the intervention group achieved significantly higher antimicrobial appropriateness scores than the control group 85.43 (80.12, 89.03) vs. 82.43 (75.54, 86.53); P = 0.004 and significantly lower total antimicrobial costs [95.16 (30.68, 180.20) vs. 185.88 (67.59, 506.08); P < 0.001] (**Table 7**).

**Table 7:** Comparison of clinical outcomes between control and intervention groups.

Indicators	Control group	Intervention group	P value
Rational use score of antibacterial drugs [M (Q1, Q3)]	82.43 (75.54, 86.53)	85.43 (80.12, 89.03)	0.004
Total antimicrobial cost [M (Q1, Q3)]	185.88 (67.59, 506.08)	95.16 (30.68, 180.20)	<0.001

M = Median; Q1 = First Quartile; Q3 = Third Quartile.

Subgroup analyses indicated higher scores in the intervention group for the Right Patient and Right Time dimensions (both P < 0.01), suggesting that pharmacist involvement particularly improved patient selection and timing of administration. No significant differences were observed for Right Drug, Right Dose, or Right Route (**Table 8**).

**Table 8:** Comparison of scores in different subgroups between control and intervention groups.

Indicators		M (Q1, Q3)	Mean ± SD	Range (Min, Max)	P value
Right Patient	Control group	12.51 (9.98, 12.51)	11.18 ± 2.10	4.17, 12.51	0.001
	Intervention group	12.51 (12.51, 12.51)	11.92 ± 1.50	4.17, 12.51	
Right Drug	Control group	25.01 (17.71, 25.01)	20.88 ± 6.87	4.67, 25.01	0.777
	Intervention group	25.01 (14.42, 25.01)	20.50 ± 7.13	0.00, 25.01	
Right Dose	Control group	25.01 (25.01, 25.01)	21.93 ± 8.18	0.00, 25.01	0.348
	Intervention group	25.01 (25.01, 25.01)	23.10 ± 6.47	0.00, 25.01	
Right Time	Control group	8.66 (5.66, 12.51)	8.40 ± 3.67	1.81, 12.51	0.000
	Intervention group	12.51 (8.54, 12.51)	10.42 ± 2.85	4.01, 12.51	
Right Route	Control group	18.75 (18.75,18.75)	18.75 ± 0.00	18.75, 18.75	1.000
	Intervention group	18.75 (18.75, 18.75)	18.75 ± 0.00	18.75, 18.75	

M, Median; Q1, First Quartile; Q3, Third Quartile.

## 4 Discussion

### 4.1 Construction of evaluation system

During system development, the 5R principles were integrated with the Delphi and AHP methods to ensure a clinically grounded indicator hierarchy and transparent weight allocation. Compared with prior work, this system extends the 5R framework from broad domains to actionable, measurable secondary and tertiary indicators with quantified weights, enabling standardized assessment across the antimicrobial prescribing process.

### 4.2 Preliminary applicability assessment and practical implications

Clinical applicability assessment demonstrated that the pharmacist-led intervention group outperformed the routine-care group in both key outcomes: higher appropriateness scores (P = 0.004) and lower total antimicrobial costs (P < 0.001). This finding aligns with previous evidence that pharmacist-led antimicrobial stewardship can improve prescribing quality and reduce costs (He et al., 2019, 2025; Xin et al., 2025). Subgroup results suggested the largest improvements occurred in the Right Patient and Right Time domains, indicating that pharmacists may be particularly effective in strengthening indication assessment and administration timing.

No significant differences were found between the two groups in the domains of Right Drug, Right Dose, and Right Route. These domains carry the greatest weight in our evaluation system. However, this high weighting reflects expert consensus on their importance rather than an expectation that pharmacist intervention will necessarily yield measurable improvements. At our institution, prescribing for Right Drug and Right Dose follows well-established national guidelines and clinical pathways. Consequently, baseline compliance in these areas was already high, leaving limited opportunity for pharmacist-led enhancements. Previous studies have also shown no differences in antimicrobial selection and dosing between groups before and after intervention in orthopedic perioperative care (Fésüs et al., 2021). For the Right Route domain, both groups performed well in matching the route to the patient and the drug. All patients received the corresponding scores. However, both groups did not receive intravenous to oral conversion therapy, so neither group scored on this tertiary indicator. Therefore, the scores in this domain were the same for both groups, with no difference. This finding reflects a common clinical practice in China. Both physicians and patients tend to believe that intravenous therapy is more effective than oral administration (Zhang et al., 2024). This perception has become a notable weakness in current clinical practice and represents an area where pharmacist-led stewardship needs to be strengthened. In the future, pharmacists should play a more active role in promoting timely intravenous therapy to oral conversion. And we should also implement standardized protocols to address this gap.

The application of this evaluation system helps identify specific practice gaps in current clinical workflows. It reveals how the pharmacist-led standardized antimicrobial pathway performs differently across the five dimensions. Clinicians can use this system to prioritize intervention domains, particularly focusing on optimizing timing of antimicrobial initiation and promoting appropriate intravenous-to-oral conversion. Pharmacists can also use the scoring results to provide targeted feedback and guide individualized antimicrobial optimization.The system had significant impact on Right Patient and Right Time, while Right Drug and Right Dose showed limited room for improvement due to high baseline compliance. Notably, the system detected a common weakness in Right Route, specifically the lack of timely conversion from intravenous to oral therapy. Future research should apply this system in multicenter settings. This will further validate its ability to guide targeted interventions and improve prescribing quality across different clinical environments.

### 4.3 Limitations and future directions

This study has limitations. First, both Delphi rounds were conducted online; despite providing detailed instructions, expert interpretation of complex indicators may still have varied.

Second, the clinical applicability assessment used a retrospective, non-concurrent cohort design, which may introduce time-related confounding. To assess this confounding, we reviewed institutional records for major stewardship policy changes, perioperative guideline updates, or formulary modifications between August 2022 and October 2024. No substantial changes were identified that would be expected to meaningfully bias comparisons between the control and intervention periods. In addition, this retrospective design brings another limitation. We were unable to gather complete data on key clinical outcomes such as the 30-day surgical site infection rate after patient discharge. Future prospective trials should incorporate clinical endpoints to fully verify the effectiveness of this evaluation system.

Third, due to the limitations of the hospital’s electronic medical record system, some clinical factors that significantly affect antimicrobial use were not included in the baseline comparison. These factors include wound contamination grade and the time from injury to emergency debridement. To minimize this impact, we collected as much available risk assessment information as possible. For example, we included injury site and surgical incision type in the baseline analysis. Given the consistent direction and magnitude of the observed differences in the primary outcomes, it is unlikely that the results are solely explained by unmeasured confounders.

Additionally, the preliminary applicability assessment samples were sourced from a single-center hospital’s hand and foot surgery department, with a limited sample size. This restricts the system’s applicability across medical institutions of different levels and regions.

Although the study was conducted in a single center, the indicator system is based on widely accepted antimicrobial stewardship principles and guideline-based practices, which supports its potential applicability in other clinical settings. Future studies can further improve this system. Multicenter and large-scale investigations will be conducted to further verify its effectiveness. Refined and stratified assessment modules will be developed, including special subgroups for open fractures. Modules tailored to different clinical stages such as prevention and treatment will also be constructed to guarantee clinical accuracy and broader generalizability.

## 5 Conclusion

In this study, we developed a 5R-guided evaluation system for appropriate antimicrobial use in open extremity trauma using a hybrid Delphi–AHP approach. With participation from 42 experts across six provinces, the final system included 5 primary, 11 secondary, and 22 tertiary indicators with validated weights. Preliminary applicability assessment suggested that applying the system within a pharmacist-led stewardship workflow can improve appropriateness scores and reduce antimicrobial costs. This evaluation system provides a standardized tool for antimicrobial use assessment in open extremity trauma. It helps identify practice gaps and supports targeted stewardship interventions.

## Data Availability

The raw data supporting the conclusions of this article are available from the corresponding author upon reasonable request. Requests to access these datasets should be directed to dengjiayu@jlu.edu.cn.
