# Supplementary material for: Development and applicability assessment of a 5R-principle-guided evaluation system for antimicrobial stewardship in open extremity trauma using a hybrid Delphi–analytic hierarchy process model
Source: Front Cell Infect Microbiol. 2026 Jul 21;16:1805356. doi: 10.3389/fcimb.2026.1805356 (PMC13433157; doi:10.3389/fcimb.2026.1805356)
Supplement: Supplementary file 1 [file DataSheet1.docx]

Supplementary Material 1

# 1 Supplementary Tables

Supplementary Table 1 Evaluation indices of the first round.

**Theme A: Right Patient**

**A1** Whether the patient has indications for preventive medication.

**A1.1** High-risk factor assessment for the patient.

**A1.2** Conducting infection risk assessment for the patient.

**A2** Whether the patient has indications for therapeutic medication.

**A2.1** Whether the patient has clinical symptoms of infection.

**A2.2** Whether the patient has laboratory test evidence of infection.

**Theme B: Right Drug**

**B1** Giving priority to first-line drugs recommended by guidelines.

**B1.1** Whether the selection of preventive drugs conforms to guideline recommendations.

**B1.2** Whether the selection of empirical therapeutic drugs is correct.

**B2** Guiding medication based on drug sensitivity test results.

**B2.1** Whether biological samples are submitted for testing.

**B2.2** Whether drugs are selected based on drug sensitivity test results.

**B2.3** Whether medications are adjusted in a timely manner based on drug sensitivity test results.

**B3** Consideration of special circumstances.

**B3.1** Drug selection for patients with drug allergy history.

**B3.2** Drug selection for patients with hepatic or renal insufficiency.

**B4** Selecting drugs with appropriate specifications.

**B4.1** Whether the drug specification matches the dosage prescribed in the medical order.

**Theme C: Right Dose**

**C1** Implementation of routine dosage.

**C1.1** Whether the dosage of antibacterial drugs used conforms to the specifications.

**C2** Individualized dosage adjustment.

**C2.1** Individualized dosage adjustment for special populations.

**C2.2** Adjustment of drug dosage based on laboratory test indicators.

**Theme D: Right Time**

**D1** Medication administration timing.

**D1.1** Whether the timing of preoperative preventive medication is reasonable.

**D1.2** Rationality of additional medication during surgery.

**D1.3** Medication administration interval compliance with instruction manual.

**D2** Medication course.

**D2.1** Whether the duration of preventive medication meets the requirements of guidelines.

**D2.2** Whether the course of therapeutic medication conforms to the specifications in the instruction manual.

**Theme E: Right Route**

**E1** Selection of medication administration route.

**E1.1** Whether the medication administration route is selected based on the actual condition of the patient.

**E2** Conversion of medication administration route.

**E2.1** Whether the medication administration route is converted in a timely manner according to the change of the patient's condition.

Supplementary Table 2 Judgment criteria and degree of influence.

| Judgment criteria | Degree of influence | | |
| --- | --- | --- | --- |
|  | High | Middle | Small |
| Theoretical basis | 0.3 | 0.2 | 0.1 |
| Clinical practice experience | 0.5 | 0.4 | 0.3 |
| Consult domestic and foreign materials | 0.1 | 0.1 | 0.1 |
| Intuitive feeling | 0.1 | 0.1 | 0.1 |

Supplementary Table 3 Familiarity score quantification table.

| Familiarity | Very familiar | familiar | Relatively familiar | Generally familiar | Unfamiliar |
| --- | --- | --- | --- | --- | --- |
| Grade | 5 | 4 | 3 | 2 | 1 |
| Quantified value | 0.9 | 0.7 | 0.5 | 0.3 | 0.1 |

Supplementary Table 4 Saaty relative importance rank table.

| Saaty Scale Value | Difference in Mean Importance Scores (X-Y) | Saaty Scale Value (Reciprocal) | Difference in Mean Importance Scores (X-Y) | Linguistic Interpretation |
| --- | --- | --- | --- | --- |
| 1 | X-Y=0.0 | 1 |  | Equal Importance |
| 2 | 0.00<X-Y≦0.25 | 1/2 | -0.25<X-Y≦0.00 |  |
| 3 | 0.25<X-Y≦0.50 | 1/3 | -0.50<X-Y≦-0.25 | Slightly More Important |
| 4 | 0.50<X-Y≦0.75 | 1/4 | -0.75<X-Y≦-0.50 |  |
| 5 | 0.75<X-Y≦1.00 | 1/5 | -1.00<X-Y≦-0.75 | Moderately More Important |
| 6 | 1.00<X-Y≦1.25 | 1/6 | -1.25<X-Y≦-1.00 |  |
| 7 | 1.25<X-Y≦1.50 | 1/7 | -1.50<X-Y≦-1.25 | Strongly More Important |
| 8 | 1.50<X-Y≦1.75 | 1/8 | -1.75<X-Y≦-1.50 |  |
| 9 | X-Y>1.75 | 1/9 | X-Y<11.75 | Extremely More Important |

Note: X and Y represent the mean importance scores of two different indicators within the same hierarchy level. The Saaty scale value is based on the calculated difference (X - Y).

Supplementary Table 5 Information on the composition of the Delphi expert panel.

| Province | City | Hospital | Hospital level | Expert professional fields (n) |
| --- | --- | --- | --- | --- |
| Jilin | Changchun | First Hospital of Jilin University | Tertiary A-level | Clinical Pharmacy (2)  Hand and Foot Surgery (3)  Medical Affairs Department (1) |
|  | Changchun | Jilin Provincial People's Hospital | Tertiary A-level | Clinical Pharmacy (2)  Orthopaedics (1)  Hand and Foot Surgery (1)  Medical Affairs Department (1)  Infection Prevention and Control Department (1) |
|  | Jilin | Jilin City Chemical Hospital | Tertiary A-level | Clinical Pharmacy (1)  Orthopaedics (2) Medical Affairs Department (1) |
|  | Meihekou | Meihekou Central Hospital | Tertiary A-level | Clinical Pharmacy (1)  Orthopaedics (2)  Hand and Foot Surgery (1) |
|  | Siping | Siping Central People's Hospital | Tertiary A-level | Clinical Pharmacy (2) |
|  | Tonghua | Tonghua Central Hospital | Tertiary A-level | Clinical Pharmacy (3)  Orthopaedics (2) Medical Affairs Department (1) |
|  | Yanji | Affiliated Hospital of Yanbian University (Yanbian Hospital) | Tertiary A-level | Clinical Pharmacy (3)  Orthopaedics (2)  Infection Prevention and Control Department (1) Medical Affairs Department (1) |
| Shandong | Qingdao | Qingdao Public Health Clinical Center (QPHCC) | Tertiary specialized hospital | Clinical Pharmacy (1) |
|  | Jinan | Jinan Maternal and Child Health Hospital | Tertiary A-level | Clinical Pharmacy (1) |
| Shanxi | Shanxi | The Second Hospital of Shanxi Medical University | Tertiary A-level | Clinical Pharmacy (1) |
| Beijing | Beijing | Beijing Friendship Hospital, Capital Medical University | Tertiary A-level | Clinical Pharmacy (1) |
| Xinjiang | Xinjiang | The First Affiliated Hospital of Xinjiang Medical University | Tertiary A-level | Clinical Pharmacy (1) |
| Hunan | Changsha | Xiangya Hospital of Central South University | Tertiary A-level | Clinical Pharmacy (1) |

Supplementary Table 6 Consensus results of the evaluation indicators from the first round.

| Clinical question | | Mean score | Coefficient of variation  (%) | Full - score frequency  (%) | Excluded |
| --- | --- | --- | --- | --- | --- |
| **Theme A: Right Patient** | | - | - | - | - |
| **A1** | Whether the patient has indications for preventive medication. | 4.76 | 0.11 | 80.95 | NO |
|  | **A1.1** High-risk factor assessment for the patient. | 4.45 | 0.19 | 54.76 | NO |
|  | **A1.2** Conducting infection risk assessment for the patient. | 4.55 | 0.11 | 54.76 | NO |
| **A2** | Whether the patient has indications for therapeutic medication. | 4.79 | 0.09 | 78.57 | NO |
|  | **A2.1** Whether the patient has clinical symptoms of infection. | 4.50 | 0.20 | 64.29 | NO |
|  | **A2.2** Whether the patient has laboratory test evidence of infection. | 4.43 | 0.25 | 61.90 | NO |
| **Theme B: Right Drug** | | - | - | - | - |
| **B1** | Giving priority to first-line drugs recommended by guidelines. | 4.55 | 0.12 | 57.14 | NO |
|  | **B1.1** Whether the selection of preventive drugs conforms to guideline recommendations. | 4.67 | 0.10 | 66.67 | NO |
|  | **B1.2** Whether the selection of empirical therapeutic drugs is correct. | 4.38 | 0.16 | 50.00 | NO |
| **B2** | Guiding medication based on drug sensitivity test results. | 4.60 | 0.19 | 71.43 | NO |
|  | **B2.1** Whether biological samples are submitted for testing. | 4.50 | 0.17 | 64.29 | NO |
|  | **B2.2** Whether drugs are selected based on drug sensitivity test results. | 4.60 | 0.19 | 69.05 | NO |
|  | **B2.3** Whether medications are adjusted in a timely manner based on drug sensitivity test results. | 4.50 | 0.13 | 54.76 | NO |
| **B3** | Consideration of special circumstances. | 4.38 | 0.15 | 47.62 | NO |
|  | **B3.1** Drug selection for patients with drug allergy history. | 4.64 | 0.12 | 69.05 | NO |
|  | **B3.2** Drug selection for patients with hepatic or renal insufficiency. | 4.57 | 0.13 | 61.90 | NO |
| **B4** | Selecting drugs with appropriate specifications. | 4.21 | 0.23 | 45.25 | NO |
|  | **B4.1** Whether the drug specification matches the dosage prescribed in the medical order. | 4.07 | 0.29 | 42.86 | YES |
| **Theme C: Right Dose** | | - | - | - | - |
| **C1** | Implementation of routine dosage. | 4.64 | 0.10 | 64.29 | NO |
|  | **C1.1** Whether the dosage of antibacterial drugs used conforms to the specifications. | 4.62 | 0.12 | 64.29 | NO |
| **C2** | Individualized dosage adjustment. | 4.55 | 0.14 | 61.90 | NO |
|  | **C2.1** Individualized dosage adjustment for special populations. | 4.52 | 0.14 | 59.52 | NO |
|  | **C2.2** Adjustment of drug dosage based on laboratory test indicators. | 4.48 | 0.14 | 54.76 | NO |
| **Theme D: Right Time** | | - | - | - | - |
| **D1** | Medication administration timing. | 4.76 | 0.09 | 76.19 | NO |
|  | **D1.1** Whether the timing of preoperative preventive medication is reasonable. | 4.69 | 0.10 | 69.05 | NO |
|  | **D1.2** Rationality of additional medication during surgery. | 4.55 | 0.13 | 59.52 | NO |
|  | **D1.3** Medication administration interval compliance with instruction manual. | 4.40 | 0.15 | 50.00 | NO |
| **D2** | Medication course. | 4.52 | 0.12 | 54.76 | NO |
|  | **D2.1** Whether the duration of preventive medication meets the requirements of guidelines. | 4.48 | 0.14 | 54.76 | NO |
|  | **D2.2** Whether the course of therapeutic medication conforms to the specifications in the instruction manual. | 4.33 | 0.16 | 45.24 | NO |
| **Theme E: Right Route** | | - | - | - | - |
| **E1** | Selection of medication administration route. | 4.48 | 0.19 | 57.14 | NO |
|  | **E1.1** Whether the medication administration route is selected based on the actual condition of the patient. | 4.62 | 0.11 | 61.90 | NO |
| **E2** | Conversion of medication administration route. | 4.29 | 0.15 | 38.10 | NO |
|  | **E2.1** Whether the medication administration route is converted in a timely manner according to the change of the patient's condition. | 4.45 | 0.15 | 54.76 | NO |

Supplementary Table 7 Consensus results of the evaluation indicators from the second round.

| Clinical question | | Mean score | Coefficient of variation  (%) | Full - score frequency  (%) | Consensus |
| --- | --- | --- | --- | --- | --- |
| **Theme A: Right Patient** | | - | - | - | - |
| **A1** | Whether the patient has indications for preventive medication. | 4.71 | 0.12 | 76.19 | YES |
|  | **A1.1** High-risk factor assessment for the patient. | 4.38 | 0.16 | 50.00 | YES |
|  | **A1.2** Performing infection risk stratification for the patient. | 4.31 | 0.15 | 40.48 | YES |
| **A2** | Whether the patient has indications for therapeutic medication. | 4.67 | 0.11 | 69.05 | YES |
|  | **A2.1** Whether the patient has clinical symptoms of infection. | 4.50 | 0.16 | 59.52 | YES |
|  | **A2.2** Whether the patient has laboratory test evidence of infection. | 4.43 | 0.19 | 59.52 | YES |
| **Theme B: Right Drug** | | - | - | - | - |
| **B1** | Giving priority to first-line drugs recommended by guidelines or specifications. | 4.62 | 0.13 | 66.67 | YES |
|  | **B1.1** Whether the selection of preventive drugs conforms to the evidence-based recommendations in current guidelines and specifications. | 4.60 | 0.12 | 61.90 | YES |
|  | **B1.2** Whether the selection of empirical therapeutic drugs is correct. | 4.19 | 0.17 | 35.71 | YES |
| **B2** | Guiding medication based on drug sensitivity or NGS test results. | 4.52 | 0.16 | 61.90 | YES |
|  | **B2.1** Whether biological samples are submitted for testing. | 4.62 | 0.13 | 66.67 | YES |
|  | **B2.2** Whether drugs are selected based on drug sensitivity or NGS test results. | 4.43 | 0.17 | 57.14 | YES |
|  | **B2.3** Whether medications are adjusted in a timely manner if empirical drugs are inconsistent with test results. | 4.26 | 0.24 | 52.38 | YES |
| **B3** | Consideration of special circumstances. | 4.19 | 0.18 | 38.10 | YES |
|  | **B3.1** Drug selection for patients with drug allergy history. | 4.71 | 0.11 | 73.81 | YES |
|  | **B3.2** Drug selection for patients with hepatic or renal insufficiency. | 4.48 | 0.15 | 57.14 | YES |
| **Theme C: Right Dose** | | - | - | - | - |
| **C1** | Implementation of routine dosage. | 4.33 | 0.16 | 45.24 | YES |
|  | **C1.1** Whether the dosage of antibacterial drugs used conforms to the specifications. | 4.67 | 0.13 | 73.81 | YES |
| **C2** | Individualized dosage adjustment. | 4.29 | 0.25 | 54.76 | YES |
|  | **C2.1** Individualized dosage adjustment for special populations. | 4.45 | 0.15 | 54.76 | YES |
|  | **C2.2** Adjustment of drug dosage based on laboratory test indicators. | 4.43 | 0.17 | 54.76 | YES |
| **Theme D: Right Time** | | - | - | - | - |
| **D1** | Medication administration timing. | 4.64 | 0.11 | 66.67 | YES |
|  | **D1.1** Whether the timing of preoperative preventive medication is reasonable. | 4.64 | 0.11 | 66.67 | YES |
|  | **D1.2** Rationality of additional medication during surgery. | 4.38 | 0.16 | 47.62 | YES |
|  | **D1.3** Medication administration interval compliance with instruction manual. | 4.36 | 0.17 | 50.00 | YES |
| **D2** | Medication course. | 4.45 | 0.17 | 59.52 | YES |
|  | **D2.1** Whether the duration of preventive medication meets the requirements of current guidelines and specifications. | 4.62 | 0.11 | 61.90 | YES |
|  | **D2.2** Whether the course of therapeutic medication meets the requirements of current guidelines and specifications. | 4.40 | 0.17 | 52.38 | YES |
| **Theme E: Right Route** | | - | - | - | - |
| **E1** | Selection of medication administration route. | 4.48 | 0.19 | 61.90 | YES |
|  | **E1.1** Whether the medication administration route is selected based on the actual condition of the patient. | 4.57 | 0.15 | 66.67 | YES |
|  | **E1.2** Whether the drug matching the medication administration route is selected. | 4.29 | 0.22 | 47.62 | YES |
| **E2** | Conversion of medication administration route. | 4.12 | 0.24 | 40.48 | YES |
|  | **E2.1** Whether the medication administration route is converted in a timely manner according to the change of the patient's condition. | 4.38 | 0.17 | 50.00 | YES |

Supplementary Table 8 Rationality score assignment for each index.

| Evaluation Indicators | Comprehensive weight | Percentage assignment |
| --- | --- | --- |
| A1.1 High-risk factor assessment for the patient. | 0.0556 | 5.56 |
| A1.2 Performing infection risk stratification for the patient. | 0.0278 | 2.78 |
| A2.1 Whether the patient has clinical symptoms of infection. | 0.0278 | 2.78 |
| A2.2 Whether the patient has laboratory test evidence of infection. | 0.0139 | 1.39 |
| B1.1 Whether the selection of preventive drugs conforms to the evidence-based recommendations in current guidelines and specifications. | 0.0990 | 9.90 |
| B1.2 Whether the selection of empirical therapeutic drugs is correct. | 0.0330 | 3.30 |
| B2.1 Whether biological samples are submitted for testing. | 0.0449 | 4.49 |
| B2.2 Whether drugs are selected based on drug sensitivity or NGS test results. | 0.0247 | 2.47 |
| B2.3 Whether medications are adjusted in a timely manner if empirical drugs are inconsistent with test results. | 0.0136 | 1.36 |
| B3.1 Drug selection for patients with drug allergy history. | 0.0233 | 2.33 |
| B3.2 Drug selection for patients with hepatic or renal insufficiency. | 0.0116 | 1.16 |
| C1.1 Whether the dosage of antibacterial drugs used conforms to the specifications. | 0.1667 | 16.67 |
| C2.1 Individualized dosage adjustment for special populations. | 0.0556 | 5.56 |
| C2.2 Adjustment of drug dosage based on laboratory test indicators. | 0.0278 | 2.78 |
| D1.1 Whether the timing of preoperative preventive medication is reasonable. | 0.0495 | 4.95 |
| D1.2 Rationality of additional medication during surgery. | 0.0208 | 2.08 |
| D1.3 Medication administration interval compliance with instruction manual. | 0.0131 | 1.31 |
| D2.1 Whether the duration of preventive medication meets the requirements of current guidelines and specifications. | 0.0278 | 2.78 |
| D2.2 Whether the course of therapeutic medication meets the requirements of current guidelines and specifications. | 0.0139 | 1.39 |
| E1.1 Whether the medication administration route is selected based on the actual condition of the patient. | 0.1406 | 14.06 |
| E1.2 Whether the drug matching the medication administration route is selected. | 0.0469 | 4.69 |
| E2.1 Whether the medication administration route is converted in a timely manner according to the change of the patient's condition. | 0.0625 | 6.25 |

Note: Comprehensive weight = (relative weight of primary indicators) × (relative weight of secondary indicators) × (relative weight of tertiary indicators).

Percentage assignment = Comprehensive weight×100%.
