# Supplementary material for: Development and applicability assessment of a 5R-principle-guided evaluation system for antimicrobial stewardship in open extremity trauma using a hybrid Delphi–analytic hierarchy process model
Source: Front Cell Infect Microbiol. 2026 Jul 21;16:1805356. doi: 10.3389/fcimb.2026.1805356 (PMC13433157; doi:10.3389/fcimb.2026.1805356)
Supplement: Supplementary file 2 [file DataSheet2.docx]

Supplementary Material 2

# 1 Department Qualification Statement

The Department of Hand and Foot Surgery at Lequn Campus, First Hospital of Jilin University, is a National Key Clinical Specialty and a key discipline of Jilin Province. The department has 106 inpatient beds, with an annual outpatient volume of approximately 37,000 visits and over 8,000 surgical procedures per year. The clinical team includes 23 physicians and 28 nurses, with 2 doctoral supervisors and 8 master's supervisors.

The department specializes in the treatment of vascular, nerve, tendon, bone, joint, and soft tissue defects of the extremities using microsurgical techniques. The scope includes open traumatic injuries of the extremities, fracture-dislocations of the hand and foot, and soft tissue reconstruction.

As the primary completing institution, the department has received multiple national and provincial awards. These include a second prize of the National Science and Technology Progress Award and two first prizes of the Jilin Provincial Science and Technology Progress Award. This information supports our study's clinical validation and confirms the department's qualification to conduct antimicrobial stewardship research in open extremity trauma.

**Pharmacist-Led Standardized Medication Therapy Pathway for Antimicrobial Stewardship in Open Extremity Trauma**

Since October 2023, the Department of Hand and Foot Surgery at Lequn Campus, First Hospital of Jilin University, has implemented a standardized, pharmacist-led antimicrobial stewardship pathway referred to as the “Five-Component Standardized Medication Therapy Pathway.” From the day of admission, all patients in the intervention group received protocol-based pharmacist-led interventions throughout their hospitalization.

The pharmacist provided clinicians with standardized protocols for safe and effective antimicrobial use based on laboratory test results and national guidelines. All interventions were delivered by uniformly trained clinical pharmacists following a predefined protocol to ensure consistency and reproducibility across patients.

(1) Daily ward round interventions

The pharmacist participated in daily clinical rounds and provided real-time recommendations on the appropriateness of antimicrobial prescriptions based on patient-specific clinical data

(2) Prescription review and optimization

The pharmacist regularly reviewed antimicrobial orders. When inappropriate or irrational prescribing practices were identified, the pharmacist communicated with treating physicians in a timely manner to optimize antimicrobial use

(3) Continuous monitoring of safety and pathogen results

The pharmacistsystematically reviewed infection-related indicators, as well as hepatic and renal function, on a daily basis to assess treatment effectiveness and detect potential adverse drug reactions to assess treatment efficacy and detect potential adverse reactions. The pharmacist also reviewed daily pathogen test results to facilitate timely transition from empirical to targeted antimicrobial therapy based on microbiological evidence

(4) Routine education and training

The pharmacist provided structured and regular training sessionsfor healthcare personnel on rational antimicrobial use, standardized microbial specimen collection, and related knowledge.

(5) Case consultation and discussion

The pharmacist regularly organized discussions on complex infection cases, provided pharmaceutical perspectives, and worked with physicians to develop optimal treatment plans.

This structured, protocol-driven pathway ensured that all patients in the intervention group received a consistent level of pharmacist-led antimicrobial stewardship, regardless of admission time or individual provider differences.

# 2 Supplementary Tables

Supplementary Table 9 Scoring criteria for each indicator of the antimicrobial rational use evaluation system.

**Theme A: Right Patient**

**A1** **Whether the patient has indications for preventive medication.**

The following scoring criteria apply to both indicators A1.1 and A1.2:

(1) Patients who did not receive prophylactic antimicrobials:

If the patient meets none of the criteria A1.1 or A1.2, this indicator is judged as "not applicable".

If the patient meets any indication for prophylactic antimicrobial use, the corresponding indicator is judged as "×".

(2) Patients who received prophylactic antimicrobials:

For patients with open extremity trauma, the surgical site may be contaminated before surgery. Guidelines recommend prophylactic antimicrobial use in this setting. Therefore, this indicator is uniformly judged as "√".

**A1.1 High-risk factor assessment for the patient. (5.56)**

Risk factors for infection include advanced age, diabetes, immunocompromised status (especially organ transplant recipients), malnutrition, and smoking.

**A1.2 Conducting infection risk assessment for the patient. (2.78)**

Whether prophylactic antimicrobials should be used perioperatively depends on multiple factors, including surgical incision type, surgical trauma severity, potential bacterial contaminants, operation duration, infection risk and consequence severity, evidence-based data on prophylaxis effectiveness, impact on bacterial resistance, and cost evaluation.

Clean surgeries (Type I incision): Prophylaxis is generally not required but may be considered in the following situations: ① large surgical area, long duration, or increased contamination risk; ② involvement of vital organs (e.g., cranial or cardiac surgery); ③ implant placement (e.g., artificial joint replacement, cardiac pacemaker); ④ presence of high-risk factors.

Clean-contaminated surgeries (Type II incision): Prophylaxis may be used because the surgical site may contain large numbers of resident bacterial flora.

Contaminated surgeries (Type III incision): Prophylaxis is required.

**A2 Whether the patient has indications for therapeutic medication.**

The following scoring criteria apply to both indicators A2.1 and A2.2:

(1) Patients who did not receive therapeutic antimicrobials:

If the patient meets none of the indications A2.1 or A2.2, this indicator is judged as "not applicable".

If any indication for therapeutic antimicrobial use is present, the corresponding indicator is judged as "×".

(2) Patients who received therapeutic antimicrobials:

If no indication for therapeutic use is present, the indicator is judged as "×".

If any indication is present, the indicator is judged as "√".

**A2.1 Whether the patient has clinical symptoms of infection. (2.78)**

Clinical signs of infection include fever or hypothermia, wound redness or swelling, and purulent wound drainage.

**A2.2 Whether the patient has laboratory test evidence of infection. (1.39)**

Any of the following conditions indicates the need for therapeutic antimicrobials:

(1) Blood routine indicators: Elevated white blood cell count (>10×10⁹/L), or significantly elevated neutrophil ratio (>75%).

(2) Inflammatory markers: Elevated C-reactive protein (>100 mg/L), or elevated procalcitonin (>0.5 ng/mL).

(3) Pathogen test results: Positive result from drug sensitivity test or NGS.

**Theme B: Right Drug**

**B1 Giving priority to first-line drugs recommended by guidelines or specifications.**

**B1.1 Whether the selection of preventive drugs conforms to the evidence-based recommendations in current guidelines and specifications. (9.90)**

(1) Patients who did not receive prophylactic antimicrobials:

The indicator is judged as "not applicable".

(2) Patients who received prophylactic antimicrobials:

If the regimen conforms to evidence-based recommendations → "√"; if not → "×".

Evidence-based recommendations for perioperative prophylactic antimicrobials:

Prophylaxis should use a single targeted agent. Broad-spectrum antimicrobials should not be used without specific indication. First-generation or second-generation cephalosporins (e.g., cefazolin, cefuroxime) are recommended. For patients with β-lactam allergy, clindamycin or macrolides may be used.

**B1.2 Whether the selection of empirical therapeutic drugs is correct. (3.30)**

Empirical therapy before culture/sensitivity results: based on injury source, infection site, and possible resistance. Options include first-generation cephalosporins (cefazolin), clindamycin (for β-lactam allergy), or third-generation cephalosporins (ceftriaxone) or piperacillin as clinically indicated.

Explanation of Medication Specificity for Pediatric Patients:

Due to immature liver and kidney function, aminoglycosides, vancomycin, norvancomycin, and chloramphenicol should be avoided.

In neonates, tetracyclines, quinolones, sulfonamides, and furan-class antibiotics are contraindicated due to severe adverse reactions.

Tetracyclines can cause tooth discoloration and enamel hypoplasia; avoid use in children under 8 years.

Quinolones should be avoided in minors because of potential bone development effects.

If the regimen conforms to the recommendations → "√"; if not → "×".

**B2 Guiding medication based on drug sensitivity or NGS test results.**

In principle, the selection of antimicrobials should be determined based onpathogen type and drug sensitivity results. For patients clinically diagnosed with bacterial infection, appropriate specimens should be collected in a timely manner for pathogen testing to identify the pathogen and drug sensitivity results, and the antimicrobial treatment regimen should be adjusted accordingly.

**B2.1 Whether biological samples are submitted for testing. (4.49)**

Antimicrobials are managed under a tiered classification system in clinical practice.

(1) Use of special-grade antimicrobials:

These agents have significant or serious adverse effects and should not be used without strong justification. Biological samples must be submitted for drug sensitivity testing before use.

If biological samples are submitted → "√"

If no sample is submitted → "×"

(2) Use of restricted-grade antimicrobials:

If the patient has clear clinical signs of infection but no biological sample is submitted → "×"

Otherwise (i.e., infection signs present with sample submitted, or no infection signs) → "√"

(3) Use of non-restricted-grade antimicrobials:

Regardless of whether a sample is submitted → "√"

**B2.2 Whether drugs are selected based on drug sensitivity or NGS test results. (2.47)**

(1) When drug sensitivity testing is not required:

Based on the result of B2.1, if no biological sample was submitted, this indicator is judged as "not applicable".

(2) Any of the following situations is judged as "√":

The patient has no clinical signs of infection and laboratory indicators are all normal, so no treatment adjustment is needed.

The test results show that the current antimicrobial is sensitive to the pathogen and effectively covers the infection, so no adjustment is needed.

The antimicrobial regimen is escalated based on test results.

(3) The following situation is judged as "×":

The drug sensitivity results show that the current bacteria are resistant to the antimicrobial being used, and the regimen needs to be adjusted but is not adjusted, or the adjusted regimen does not match the sensitive drugs reported in the sensitivity test.

**B2.3 Whether medications are adjusted in a timely manner if empirical drugs are inconsistent with test results. (1.36)**

(1) The following situations are judged as "not applicable":

Based on the result of B2.1, no drug sensitivity test was performed.

Based on the result of B2.2, no adjustment of the treatment regimen is needed.

(2) When the treatment regimen needs to be adjusted based on the test results:

If the regimen is adjusted within 24 hours → "√"

If the regimen is not adjusted within 24 hours → "×"

**B3 Consideration of special circumstances.**

**B3.1 Drug selection for patients with drug allergy history. (2.33)**

(1) Patient has no drug allergy:

This indicator is judged as "not applicable".

(2) Patient has a drug allergy:

The correctness of the selected antimicrobial agent is determined based on the patient's drug allergy history.

**B3.2 Drug selection for patients with hepatic or renal insufficiency. (1.16)**

(1) Patient has normal liver and kidney function:

This indicator is judged as "not applicable".

(2) Patient with hepatic impairment:

Avoid using chloramphenicol, rifampin, and erythromycin esters.

Macrolides (excluding esters), clindamycin, and lincomycin may be used but liver function should be closely monitored.

Patient with renal impairment:

Aminoglycosides and polymyxins are contraindicated.

Sulfonamides are contraindicated in patients with severe renal failure.

If the regimen conforms to the above criteria → "√"; if not → "×".

**Theme C: Right Dose**

**C1 Implementation of routine dosage.**

**C1.1 Whether the dosage of antibacterial drugs used conforms to the specifications. (16.67)**

(1) If the patient is elderly (≥65 years), a child (≤14 years), or has hepatic or renal impairment, this indicator is judged as "not applicable".

(2) For patients in the normal population:

If the dosage conforms to the recommended dose for prophylaxis or treatment in the drug instruction manual → "√"; if not → "×".

**C2 Individualized dosage adjustment.**

**C2.1 Individualized dosage adjustment for special populations. (5.56)**

(1) Patients who are not elderly or children:

This indicator is judged as "not applicable".

(2) Elderly or pediatric patients:

For elderly patients: Does the drug package insert indicate dose reduction, and does the dosage meet the adjustment requirements? → "√"; if not → "×".

For pediatric patients: Is the dosage calculated according to the weight-based standards specified in the drug package insert? → "√"; if not → "×". (e.g., for Cefazolin Sodium for Injection: for children over 1 month old, 25-50 mg/kg/day in 3-4 divided intravenous slow injections for mild to moderate infections; up to 100 mg/kg/day for severe infections.)

**C2.2 Adjustment of drug dosage based on laboratory test indicators. (2.78)**

(1) Patients with normal liver and kidney function:

This indicator is judged as "not applicable".

(2) Patients with hepatic or renal impairment:

The degree of hepatic or renal impairment must be considered when selecting the drug dose.

If the drug is contraindicated in patients with hepatic or renal impairment → "×"

If the drug is not contraindicated, and the dose conforms to the recommended dose in the drug instruction manual → "√"; if not → "×"

**Theme D: Right Time**

**D1 Medication administration timing.**

**D1.1 Whether the timing of preoperative preventive medication is reasonable. (4.95)**

(1) Patients who did not receive prophylactic antimicrobials:

This indicator is judged as "not applicable".

(2) Patients who received preoperative prophylactic antimicrobials:

Intravenous administration should be given within 0.5 to 1 hour before skin or mucosal incision or at the start of anesthesia, with surgery beginning after the infusion is completed. For drugs requiring prolonged infusion time, such as vancomycin or fluoroquinolones, administration should begin 1 to 2 hours before surgery.

If the timing conforms to the above guideline recommendations → "√"; if not → "×".

**D1.2 Rationality of additional medication during surgery. (2.08)**

Conditions for redosing during surgery:

Redosing should be given during surgery if any of the following conditions is present: surgery duration exceeds 3 hours, or exceeds twice the half-life of the drug used, or blood loss in adults exceeds 1500 mL.

(1) Redosing given during surgery:

If any of the above conditions is present (redosing was indicated and given) → "√"

If none of the above conditions is present → "×"

(2) Redosing not given during surgery:

If none of the above conditions is present (redosing was not needed) → "√"

If any of the above conditions is present (redosing was indicated but not given) → "×"

**D1.3 Medication administration interval compliance with instruction manual. (1.31)**

Time-dependent antimicrobials (e.g., penicillins, cephalosporins and other β-lactams, erythromycin, clindamycin) should be administered multiple times daily. Concentration-dependent antimicrobials (e.g., fluoroquinolones and aminoglycosides) may be administered once daily. The specific dosing frequency should follow the drug instruction manual.

If the dosing frequency conforms to the instruction manual recommendation → "√"; if not → "×".

**D2 Medication course.**

**D2.1 Whether the duration of preventive medication meets the requirements of current guidelines and specifications. (2.78)**

(1) Patients who did not receive prophylactic antimicrobials:

This indicator is judged as "not applicable".

(2) Patients who received prophylactic antimicrobials:

The duration of perioperative prophylactic antimicrobial use is 24 hours. For contaminated surgeries or dirty-infected surgeries, the duration may be extended to 48 hours when necessary.

If the duration conforms to the above guideline recommendations → "√"; if not → "×".

**D2.2 Whether the course of therapeutic medication meets the requirements of current guidelines and specifications. (1.39)**

The duration of antimicrobial therapy varies by type of infection. In general, treatment should continue until the patient's body temperature returns to normal and clinical symptoms have resolved for 72 to 96 hours. For patients with localized lesions, treatment should continue until the infection is controlled or completely resolved. Empirical therapy should not exceed 7 days in principle. If the drug instruction manual provides a clear recommended duration, that duration should be followed.

If the duration conforms to the above criteria → "√"; if not → "×".

**Theme E: Right Route**

**E1 Selection of medication administration route.**

**E1.1 Whether the medication administration route is selected based on the actual condition of the patient. (14.06)**

(1) For prophylactic antimicrobial use:

Intravenous administration is recommended.

(2) For therapeutic antimicrobial use:

Patients with mild to moderate infection should receive oral therapy.

Intravenous administration may be given under the following special circumstances: ① inability to tolerate oral administration; ② presence of factors that clearly affect oral drug absorption; ③ the selected antimicrobial has no oral formulation available; ④ severe infection or rapid disease progression requiring urgent intravenous control; ⑤ poor patient adherence to oral medication.

If the regimen conforms to the above criteria → "√"; if not → "×".

**E1.2 Whether the drug matching the medication administration route is selected. (4.69)**

Determine whether the selected drug is optimally matched to the current route of administration.

If matched → "√". If not matched → "×"

**E2 Conversion of medication administration route.**

**E2.1 Whether the medication administration route is converted in a timely manner according to the change of the patient's condition. (6.25)**

For infected patients receiving injectable therapy, when the condition improves after initial intravenous treatment and oral administration is feasible, timely conversion from intravenous to oral therapy (sequential therapy) is judged as "√"; otherwise, it is judged as "×".

Any of the following conditions indicates that oral administration is not feasible:

① Inability to tolerate oral administration. ② Presence of factors that clearly affect oral drug absorption (e.g., gastrointestinal function not recovered). ③ Poor patient adherence to oral medication.

Note: The values in parentheses are the 100-point scale scores for each tertiary indicator. Indicators judged as "√" receive the corresponding weighted score. Indicators judged as "×" receive 0 points. Indicators judged as "not applicable" are excluded from the total score calculation.

The final appropriateness score is calculated as follows: Final score = (Sum of actual scores from "√" indicators / (100 - Sum of weights of "not applicable" indicators)) * 100.
